# Analysis of in vivo humeral rotation of reverse total shoulder arthroplasty patients during shoulder abduction on the scapular plane with a load

**DOI:** 10.1186/s42836-023-00207-1

**Published:** 2023-10-05

**Authors:** Yuji Takahashi, Naoya Nishinaka, Kanji Furuya, Takashi Ikeda, Tetsuya Jinno, Atsushi Okawa, Tomoko Sakai

**Affiliations:** 1 Department of Rehabilitation Medicine, Tokyo Medical and Dental University, 1-5-45 Yushima, Bunkyo-Ku, Tokyo, 113-8510 Japan; 2 Showa University School of Nursing and Rehabilitation Science, 1865 Tohkaichiba-Cho Midori-Ku, Yokohama, Kanagawa 226-8555 Japan; 3 Showa University Faculty of Health Care, 1865 Tohkaichiba-Cho Midori-Ku, Yokohama, Kanagawa 226-8555 Japan; 4 Showa University Research Institute for Sport and Exercise Sciences, 2-1-1 Fujigaoka Aoba-Ku, Yokohama, Kanagawa 227-8518 Japan; 5 Department of Orthopaedic Surgery, Showa University Fujigaoka Hospital, 1-30 Fujigaoka Aoba-Ku, Yokohama, Kanagawa 227-8501 Japan; 6 Department of Orthopaedic Surgery, Dokkyo Medical University Saitama Medical Center, 2-1-50 Minamikoshigaya, Koshigaya, Saitama 343-8555 Japan; 7 Department of Orthopaedic Surgery, Tokyo Medical and Dental University, 1-5-45 Yushima, Bunkyo-Ku, Tokyo, 113-8510 Japan

**Keywords:** Shoulder, Kinematics, Reverse total shoulder arthroplasty, Glenohumeral rotation, Loaded, Shoulder function

## Abstract

**Background:**

Few studies have investigated the kinematics after reverse total shoulder arthroplasty (RTSA). This study aimed to compare the shoulder kinematics in RTSA patients during shoulder abduction on the scapular plane with and without a load and yield information regarding the function of stabilizing the joints against gravity for the functional assessment of the shoulder after RTSA, which could lead to changes in postoperative rehabilitation treatment.

**Methods:**

Twenty RTSA patients (7 men, 13 women; mean age: 78.1 [64–90] years) were examined. First, active shoulder abduction in the scapular plane was captured using single-plane fluoroscopic *X*-ray images. Imaging was performed by stipulating that one shoulder abduction cycle should be completed in 6 s. Two trials were conducted: one under a load equivalent to 2% of body weight and one without a load. Next, a three-dimensional (3D) model of each humeral and scapular component was matched to the silhouette of the fluoroscopic image to estimate the 3D dynamics. By using the 3D dynamic model obtained, the kinematics of the glenosphere and humeral implant were calculated relative to the shoulder abduction angle on the scapular plane and were compared between groups with and without a load. A one-way analysis of variance and a post hoc paired *t*-test with a statistical significance level of 0.05 were performed.

**Results:**

The humeral internal rotation decreased with a load at shoulder abduction between 40° and 90° on the scapular plane (*P* < 0.01, effect size: 0.15). No significant differences in scapular upward rotation (*P* = 0.57, effect size: 0.022), external rotation (*P* = 0.83, effect size: 0.0083) and posterior tilting (*P* = 0.74, effect size: 0.013) were observed between groups with and without a load. The main effect was not observed with and without a load (*P* = 0.86, effect size: 0.0072). However, the scapulohumeral rhythm was significantly greater without a load during shoulder joint abduction between 40° and 60° on the scapular plane.

**Conclusion:**

In RTSA patients, the glenohumeral joint was less internally rotated, and the scapulohumeral rhythm decreased under loaded conditions. It was stabilized against the load through the mechanical advantage of the deltoid muscle and other muscles.

## Background

Rotator cuff-tear arthropathy [1] (CTA) is osteoarthritis of the shoulder following a massive tear of the rotator cuff and presents with symptoms such as pain, joint subluxation, and elevation failure [2]. To address the issue, reverse total shoulder arthroplasty (RTSA) can be performed, by which the glenohumeral joint is converted into a reversed ball and socket articulation. RTSA was devised by Grammont [3] in the 1980s as a method that used an artificial joint for a shoulder with a non-functioning rotator cuff. The biomechanical change medializes the center of rotation, thereby altering the moment arm of the deltoid muscle and allowing the shoulder to be elevated in the presence of a non-functioning rotator cuff [4]. Hence, the artificial shoulder joint in RTSA uses a mechanism different from that of a normal joint when elevating the arm [2], and understanding its three-dimensional (3D) kinematics may provide helpful information for improving postoperative rehabilitation and implant design.

Previous studies have investigated the changes in kinematics following the RTSA procedure [2, 5–12], with some reports evaluating scapular kinematics [5, 9] and scapulohumeral rhythm [9, 12]. Nonetheless, only a few reports have examined the glenohumeral joint kinematics. Furthermore, previous studies have reported on the shoulder joint kinematics with a load on the upper limb [13–16]. During abduction against gravity, the deltoid muscle and other tissues stabilize the glenohumeral joint and draw the humeral head to the center of the glenoid [17]. Applying a load on the upper limb not only reproduces the joint load for daily activities, such as grasping an object but also provides useful information regarding functional changes for stabilizing the glenohumeral joint by examining the difference in reaction between healthy and pathological shoulders. Some reports suggest that applying a load changes the scapulohumeral rhythm and scapular movement [13, 14], whereas other reports indicate that it does not [15, 16]. Kon et al. [14] analyzed scapular kinematics by applying a 3-kg load to the healthy shoulder and found that checking a patient’s shoulder function and scapular stability is simple. Several reports [2, 5, 12] have examined the scapulohumeral rhythm in RTSA patients under a load. Nevertheless, no study has assessed the glenohumeral joint kinematics under a load in RTSA patients. Considering that the glenohumeral joint is a structure in which the positions of the ball and socket are reversed, understanding the 3D glenohumeral joint kinematics in RTSA is important.

The present study aimed to compare the kinematics during shoulder abduction on the scapular plane in RTSA patients with and without a load and yield information regarding the function of stabilizing the joints against gravity. The information is important for the functional assessment of the shoulder after RTSA. We hypothesized that kinematics would significantly differ between RTSA patients with and without a load. If the shoulder kinematics changes with or without a load, the required muscle activity and joint mobility may differ during shoulder movement. This may be important for postoperative rehabilitation, since conventional treatments after total shoulder arthroplasty may not be appropriate for RTSA.

## Methods

This was a non-randomized, experimental, observational study.

### Participants

The inclusion criteria were as follows: Adult patients who underwent RTSA between October 2014 and April 2021, and had been monitored for more than 1 year and could raise the shoulder on the scapular plane by 90° or more. Patients who had undergone revision surgery, patients in whom *X*-ray fluoroscopy was difficult to perform, and those who had difficulty visiting the hospital because of other diseases were excluded from the study.

**Fig. 1:**
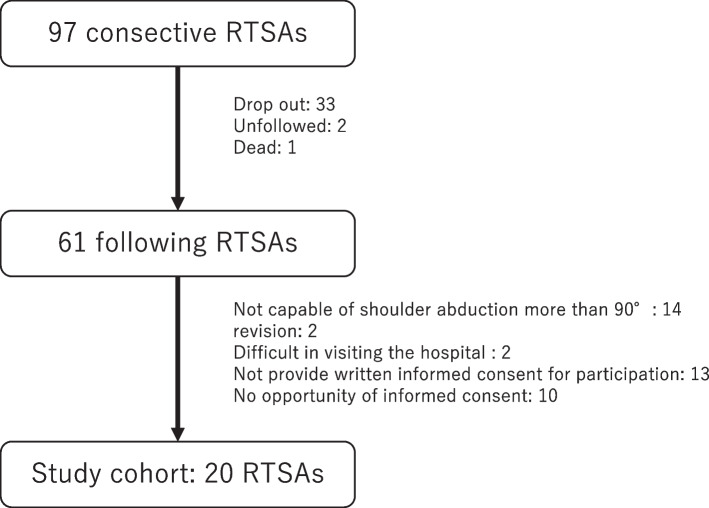
Flowchart for identification of participants

A total of 20 RTSA patients (7 men and 13 women; mean age: 78.1 [64–90] years), who provided written informed consent for participating in the project, were included in the study following Institutional Review Board approval (Fig. 1). Table 1 presents the characteristics of each participant. The mean follow-up was 41.5 (13–63) months. The RSTA involved 14 right shoulders, 4 left shoulders, and both shoulders in two cases. One patient was left-handed, and the other patients were right-handed. In 15 patients, the dominant side was operated on, while in 5 patients the non-dominant side received the procedure. In patients with both shoulders operated, the right side underwent surgery first and was thus measured. Indications for RTSA included CTA in 11 shoulders, extensive rotator cuff tear in 4 shoulders, shoulder joint osteoarthritis in 3 shoulders, and proximal humeral fracture in 2 shoulders.

**Table 1:** Characteristics of each participant

**Case No**	**Diagnosis**	**Affected side**	**Time from surgery (months)**	**Approach**
1	CTA	Right	41	A-S
2	Osteoarthritis	Right	62	D-P
3	Proximal humeral fracture	Left	50	D-P
4	Extensive rotator cuff tear	Right	51	A-S
5	CTA	Right	63	D-P
6	Proximal humeral fracture	Right	30	A-S
7	CTA	Right	55	D-P
8	CTA	Left	72	D-P
9	CTA	Left	23	D-P
10	CTA	Right	30	A-S
11	CTA	Right	34	D-P
12	CTA	Both shoulder (Right)	14	D-P
13	CTA	Right	21	D-P
14	Extensive rotator cuff tear	Right	13	A-S
15	Extensive rotator cuff tear	Right	22	A-S
16	CTA	Both shoulder (Right)	47	D-P
17	Osteoarthritis	Right	33	D-P
18	Extensive rotator cuff tear	Left	55	D-P
19	Osteoarthritis	Right	63	D-P
20	CTA	Right	51	A-S

*CTA* Cuff-tear arthropathy, *D-P* Deltopectoral approach, *A-S* Anterosuperior approach

Equinoxe (Exactech, Gainesville, FL, USA) was used in 10 cases, in which 36-mm and 38-mm glenospheres were inserted in seven and three cases, respectively. Aequalis Reversed (Tornier, Saint Martin, France) was utilized in seven cases, in which 36-mm glenospheres were inserted in all cases. Lastly, SMR Reversed (Lima, Tokyo, Japan) was employed in three cases, in which 36-mm glenospheres were inserted in all cases. In all patients, the teres minor muscle was still intact and retained.

### Clinical assessment

Patients were clinically examined at the time of fluoroscopic X-ray imaging. We evaluated patients’ active and passive range of motion using a goniometer, the American Shoulder and Elbow Surgeons (ASES) Shoulder Score, and the Constant Score [18].

### Imaging of joint dynamics

Single-plane fluoroscopic X-ray imaging was performed under the supervision of a senior surgeon. The participants were asked to take a sitting position, with the trunk upright, the upper limbs hanging down, and the palm facing forward in a thumb-up position. Shoulder abduction was performed two times on the scapular plane, defined as the plane where the scapular spine aligns with the humeral shaft. One movement cycle comprised lifting the arm from the arm-on-the-side position to the maximum abducted position and returning to the arm-on-the-side position. The movement had to be completed within 6 s, which was monitored by using a watch. We analyzed the finding on the second attempt, and for participants with frame-out movement on the second attempt, the finding on the first attempt was analyzed. Two trials were performed: one under a load (a weight equivalent to 2% of body weight wrapped around the distal forearm) and one without a load.

### Generation of a 3D model of the artificial shoulder joint

**Fig. 2:**
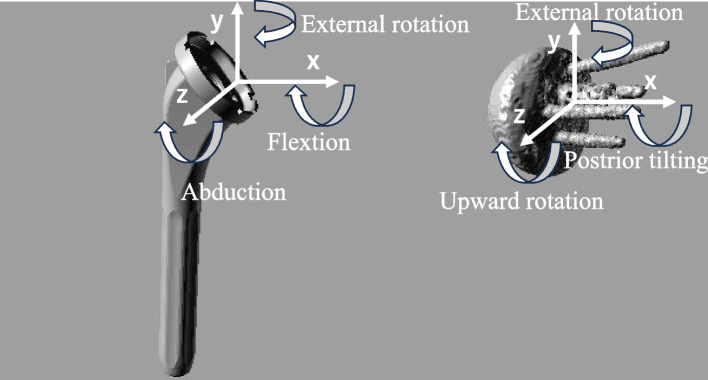
The humeral and scapular component embedded coordinate system and definition of rotations

A 3D model of the humeral component in each participant was created on the basis of the computer-aided design model. A 3D model of the scapular component was made by using computer software (ITK snap Penn Image Computing and Science Laboratory, Philadelphia, PA, USA) based on computed tomography (CT) information [19], taken with the arm at the side position to define the screw position and angle for fitting to the contour of the fluoroscopic X-ray image. Next, a coordinate system was defined for each 3D model (Fig. 2) using computer software (Rhinoceros®; Robert McNeel & Associates, Seattle, WA, USA). The origin of the humeral component was defined as the center of the polyethylene insert; the stem axis as the y-axis; the line directing toward the highest point of the circular insert from the origin as the x-axis; the axis perpendicular to the xy-plane as the z-axis. The origin of the scapular component was defined as the center of the hemisphere; the axis of the base plate as the x-axis; the line from the center of the baseplate’s circle to the cranial vertex as the y-axis; the anteroposterior direction as the z-axis on a plane perpendicular to the x-axis.

### Two-dimensional (2D)-3D registration and data analysis

**Fig. 3:**
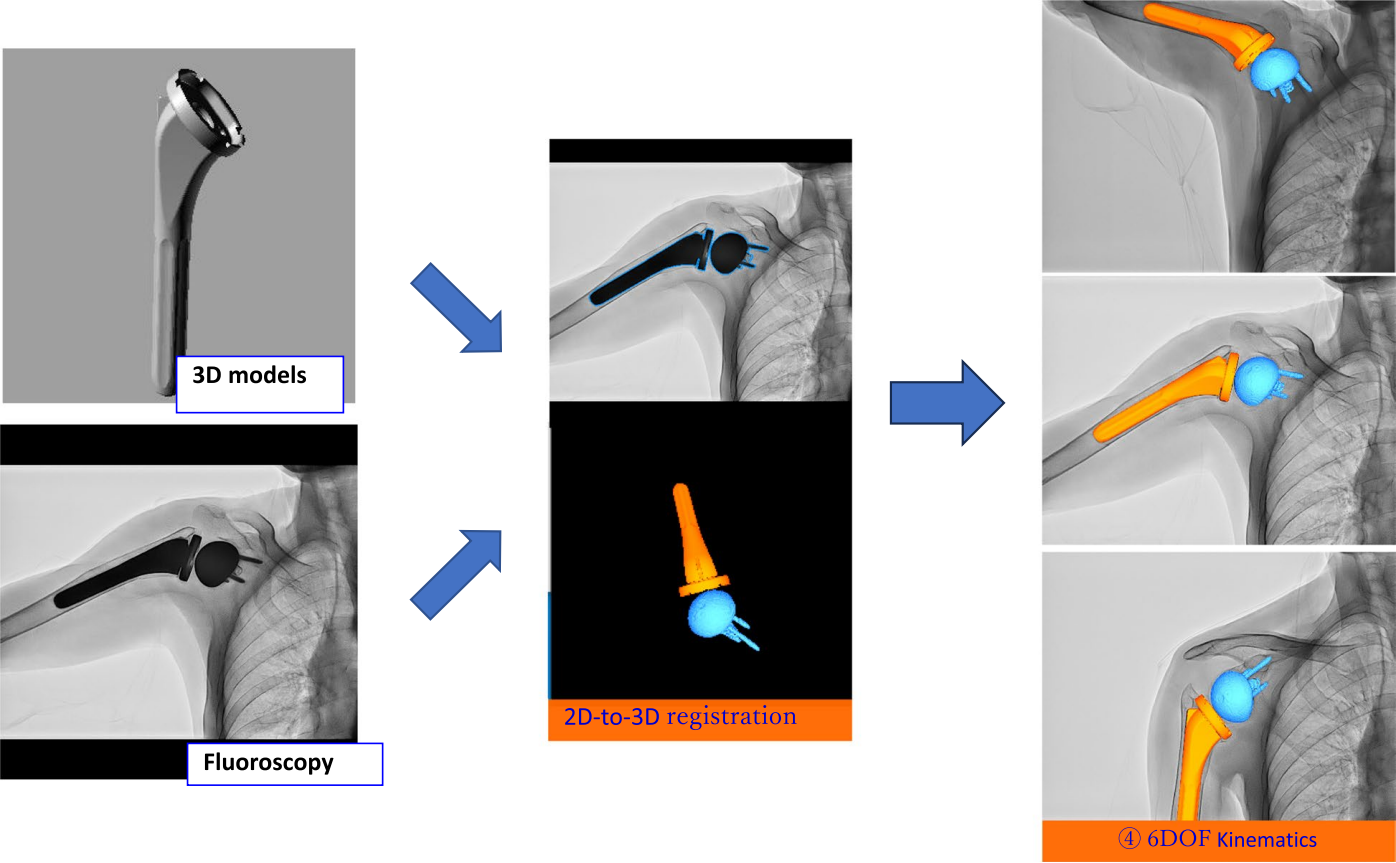
The 2-dimensional-3-dimensional model image registration in RTSA. Three-dimensional artificial models were fitted to the contour of the fluoroscopic X-ray image

Using JointTrack software [20] (www.sourceforge.net/projects/jointtrack), the 3D model of the artificial joint was fitted to the contour of the fluoroscopic X-ray image, and the 3D position was estimated (Fig. 3). The implant kinematics relative to the X-ray coordinate system and the kinematics of a humeral component relative to a scapular component were determined using the Euler angle. The shoulder abduction angle on the scapular plane was defined as the rotation angle of the humeral component on the z-axis. The rotation angle of the glenohumeral joint was defined as the rotation angle of the humeral component on the y-axis relative to the origin of the scapular component along the y-axis of the scapular component. The rotation angle of the scapular component was defined as follows: anterior/posterior tilting on the x-axis; internal/external rotation on the y-axis; and upward/downward rotation on the z-axis. The scapulohumeral rhythm was the ratio of the glenohumeral abduction angle to the scapular upward rotation angle. ΔHumerus (ΔH) was defined as an increment in shoulder abduction angle between the reference point and the next 10°, and the value is constantly 10°. Data analysis was performed using the 10° interval of shoulder abduction as the basic unit. ΔScapula (ΔS) was defined as an increment of scapular upward rotation angle, calculated for every 10° of shoulder abduction. The glenohumeral abduction angle was represented by the difference between ΔH and ΔS, and the scapulohumeral rhythm was calculated as (ΔH – ΔS) / ΔS [10, 12].

### Accuracy of the 2D-3D registration technique

Intra- and interobserver reproducibility of the 2D-3D registration technique was assessed using the standard deviation of the glenohumeral rotation angle and scapular component rotation angle obtained from the reconstruction of each subject that defined the 95% confidence interval. Our study included 12 participants, and each participant was evaluated by the same examiner (a physical therapist) on two test days, with a test-to-test interval of 7 days. Additionally, the participants were evaluated by a second examiner (a senior surgeon) on the second test day to assess interrater reliability.

### Statistical analysis

A one-way analysis of variance was performed for statistical analysis of each joint angle, with the presence or absence of a load as a factor to inspect the hypothesis that “shoulder kinematics would significantly differ between RTSA patients with and without a load.” Multiple comparison testing was conducted using a paired *t*-test with Bonferroni correction. Statistical analysis was performed using JMP®16.0 (SAS Institute Inc., Cary, NC, USA) with a statistical significance level set at 0.05. The required sample size was calculated to be 199 when the effect size was 0.1, the significance level (alpha error probability) was 0.05, and the power (1-beta error probability) was 0.8. In addition, we calculated the correlation coefficient to provide a quantitative assessment.

## Results

Table 2 summarizes the clinical assessment results of participants. Intra- and interrater reliability of glenohumeral and scapular kinematics during shoulder abduction in the scapular plane showed good reliability [21], being > 0.70 for the most part. Table 3 presents the results of the correlation coefficient.

**Table 2:** Clinical assessments of participants

**Mean (± SD) of clinical evaluation**	
Passive range of motion	
Flexion (degree)	137.50 ± 15.17
Abduction (degree)	128.00 ± 19.08
External rotation (degree)	42.25 ± 18.46
Functional score	
ASES score	70.61 ± 15.53
Constant score	58.40 ± 10.52

Clinical measures (Mean ± standard deviation [SD])

**Table 3:** Correlation coefficients

	Loaded	Unloaded
Glenohumeral internal rotation	0.53	0.41
Scapular upward rotation	0.28	0.32
Scapular external rotation	0.038	0.064
Scapular postrior tilting	0.24	0.21

**Fig. 4:**
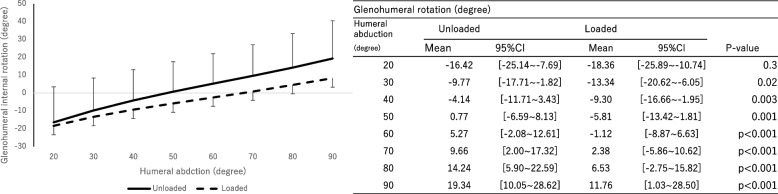
The rotation angle of the humeral component relative to scapular component. Internal ( +) / external (-) rotation. The significance level adjusted by Bonferroni correction was 0.00714. A significant difference (*P* < 0.05) was found at 40 degrees shoulder abduction

The main effect was observed in the rotation angle of the humeral component with respect to the scapular component (Fig. 4) with and without a load (*P* < 0.01, effect size: 0.15). External rotation was significantly greater with a load during shoulder joint abduction between 40° and 90° on the scapular plane.

**Fig. 5:**
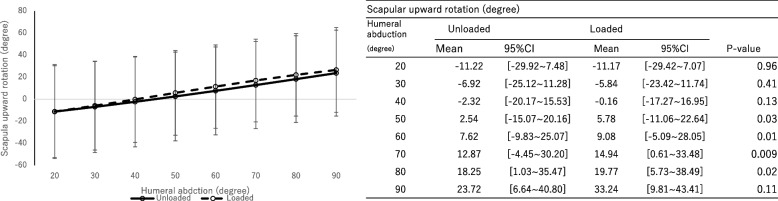
The rotation angle of the scapular component. Upward ( +) / downward ( −) rotation. The significance level adjusted by Bonferroni correction was 0.00714

**Fig. 6:**
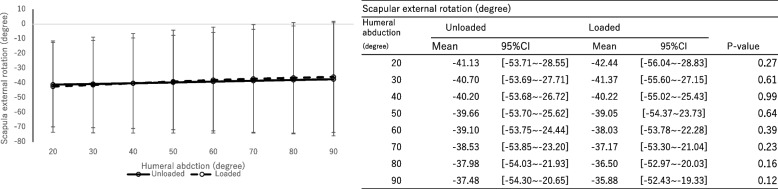
The rotation angle of the scapular component. External ( +) / internal ( −) rotation. The significance level adjusted by Bonferroni correction was 0.00714

**Fig. 7:**
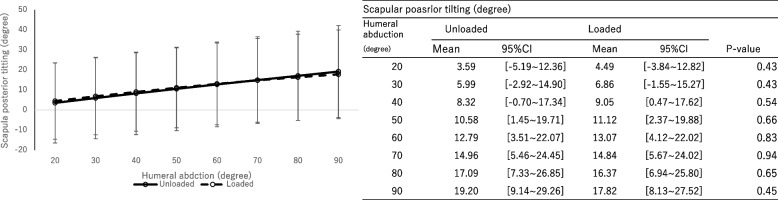
The rotation angle of the scapular component. Posterior ( +) / anterior ( −) tilting. The significance level adjusted by Bonferroni correction was 0.00714

There were no significant differences in scapular component upward rotation (Fig. 5, *P* = 0.57, effect size: 0.022), external rotation (Fig. 6, *P* = 0.83, effect size: 0.0083), and posterior tilting (Fig. 7, *P* = 0.74, effect size: 0.013) between groups with and without a load.

**Fig. 8:**
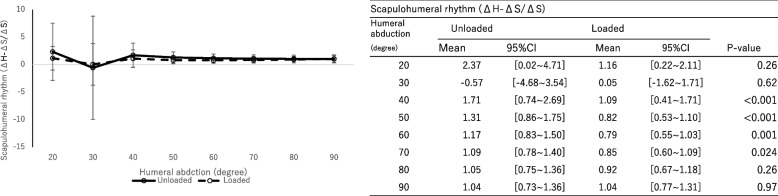
Scapulohumeral rhythm under loaded and unloaded conditions. The significance level adjusted by Bonferroni correction was 0.00714. A significant difference (*P* < 0.05) was found at 40 degrees to 60 degrees shoulder abduction

The main effect was not observed in the scapulohumeral rhythm with and without a load (Fig. 8, *P* = 0.86, effect size: 0.0072). However, the scapulohumeral rhythm was significantly greater without a load during shoulder joint abduction between 40° and 60° on the scapular plane.

## Discussion

The results of the study partially supported our hypothesis. The glenohumeral joint was less internally rotated under the loaded condition than under the unloaded condition during shoulder abduction of 40° to 90° on the scapular plane. The present study analyzed the rotation of the glenohumeral joint as a rotation of the humeral component relative to the scapular component. Therefore, the rotational angle of the glenohumeral joint was unaffected by the movement of the scapula, which is a notable strength of this study. It is possible that the influence of gravity changes as the scapula moves. However, as activities of daily life are performed under similar conditions, this study was conducted under these conditions. In contrast, no significant differences in scapulothoracic joint kinematics were observed. However, scapulohumeral rhythm decreased under loaded conditions than under unloaded conditions during arm elevation in the scapular plane. Some studies [2, 12] analyzed the kinematics with a load during shoulder abduction in RTSA patients. Walker et al. [12] examined the scapulohumeral rhythm with a 1.4-kg load during arm abduction on the frontal plane in RTSA patients and reported no difference between the loaded and unloaded conditions. The difference in the results by Walker et al. [12] might be related to the plane of shoulder abduction. Kwon et al. [2] reported that the scapulohumeral rhythm decreased when hand weights were added during arm elevation in the scapular plane. The results of the present study are consistent with the findings by Kwon et al. [2].

Based on this result, the glenohumeral joint was less internally rotated, and the scapulohumeral rhythm decreased under loaded conditions. Kon et al. [14] reported that the scapulohumeral rhythm was greater (less scapular motion) under the loaded condition in the healthy shoulder. The contrasting results by Kon et al. [14] indicated functional differences between the healthy shoulder and RTSA one. In a healthy shoulder, which was speculated to be due to holding the scapula to the torso against loads [14]. In contrast, in RTSA, the initial position on the scapula is rotated to increase the deltoid muscle length and provide a greater mechanical advantage for the deltoid muscle [2] and the scapular muscles exert a greater force to hold the scapula against loads. The range of 40° to 90° for shoulder abduction was mainly the cycle of the hanging joint [22], and bony support against the load was not available. Therefore, the glenohumeral joint was stabilized against the load through the mechanical advantage of the deltoid muscle and other muscles. Consequently, the patients might have altered the glenohumeral rotation. RTSA patients who were unable to perform shoulder joint abduction on the scapular plane under loaded may require enhanced function to mitigate scapulohumeral rhythm reduction. This main finding holds significance because it offers a screening method to evaluate shoulder function, particularly stability, in RTSA cases. For example, this insight could potentially determine whether individuals with increased internal rotation of the humeral component under load will be able to externally rotate the humeral component, including scapulohumeral rhythm, against load.

In this study, the humeral component internally rotated relative to the scapular component in RTSA patients during shoulder abduction on the scapular plane. Matsuki et al. [10] also examined these kinematics and reported that the humeral component rotated externally. Although our study showed different results, we recorded active shoulder abduction with participants assuming a sitting position. Matsuki et al. [10] did so with the participants in a standing position. Some studies [23, 24] reported that shoulder joint kinematics changed between standing and sitting positions. However, there are no reports regarding humeral rotation during arm elevation. The retroversion angle of the humeral component has been reported to affect its rotation during shoulder abduction in RTSA patients [25]. Gulotta et al. [25] showed that the internal rotation angle decreased with an increasing retroversion angle at shoulder abduction between 20° and 40° on the scapular plane. The discrepancy in the result from Matsuki et al. [10] may be ascribed to the difference in measurement positions and retroversion angle of the humeral component.

This study had several limitations. First, this study involved a relatively small number of patients. Nevertheless, given that sample size is unrealistic owing to X-ray fluoroscopy and CT imaging, the number of cases was determined using a 2D-3D registration technique based on previous reports [10, 12, 14, 17, 26]. Second, fluoroscopic images were taken on a single plane and, therefore, lacked accuracy in out-of-plane rotations, which was also mentioned by Matsuki et al. [26]. Furthermore, Bey et al. [27] reported that biplane fluoroscopy improved accuracy in out-of-plane rotations. However, because the kinematic parameters in our study were insensitive to out-of-plane translation errors, single-plane fluoroscopic imaging was adequate. It was also necessary to fully consider the indications, such as using single-plane fluoroscopy and biplane fluoroscopy as required. Finally, the variation of RTSA, implant designs, retroversion of the humeral component, surgical approaches, whether the surgical side is the dominant side or not, and measurement positions were not evaluated in this study, considering that the subject of this study was relatively good shoulder abduction on the scapular plane. Hence, an investigation including these factors should be conducted to provide more information for evaluating the shoulder function after the RTSA.

## Conclusion

This study investigated the kinematics during shoulder abduction in RTSA patients using a 2D/3D registration method. Herein, we compared the angle of rotation between the humeral and scapular components during shoulder abduction on the scapular plane under two conditions: with and without load application. At a shoulder abduction between 40° and 90° on the scapular plane, internal rotation of the humeral component was significantly less with a load than that without a load. The scapulohumeral rhythm was significantly greater without a load during shoulder joint abduction between 40° and 60° in the scapular plane. No significant differences in scapulothoracic joint kinematics were noted.

## Data Availability

The datasets generated and analyzed during the current study are available from the corresponding author on reasonable request.

## Abbreviations

RTSA: Reverse total shoulder arthroplasty
3D: Three-dimensional
CTA: Cuff-tear arthropathy
ASES: American Shoulder and Elbow Surgeons
CT: Computed tomography
2D: Two-dimensional
ΔH: ΔHumerus
ΔS: ΔScapula

## References

[1] Neer CS, Craig EV, Fukuda H (1983). Cuff-tear arthropathy. J Bone Joint Surg Am.

[2] Kwon YW, Pinto VJ, Yoon J, Frankle MA, Dunning PE, Sheikhzadeh A (2012). Kinematic analysis of dynamic shoulder motion in patients with reverse total shoulder arthroplasty. J Shoulder Elbow Surg.

[3] Grammont PM, Baulot E (1993). Delta shoulder prosthesis for rotator cuff rupture. Orthopedics.

[4] Boileau P, Watkinson DJ, Hatzidakis AM, Balg F (2005). Grammont reverse prosthesis: design, rationale, and biomechanics. J Shoulder Elbow Surg.

[5] de Toledo JM, Loss JF, Janssen TW, van der Scheer JW, Alta TD, Willems WJ (2012). Kinematic evaluation of patients with total and reverse shoulder arthroplasty during rehabilitation exercises with different loads. Clin Biomech.

[6] Alta TD, Bergmann JH, Veeger DJ, Janssen TW, Burger BJ, Scholtes VA (2011). Kinematic and clinical evaluation of shoulder function after primary and revision reverse shoulder prostheses. J Shoulder Elbow Surg.

[7] Alta TD, de Toledo JM, Veeger HE, Janssen TW, Willems WJ (2014). The active and passive kinematic difference between primary reverse and total shoulder prostheses. J Shoulder Elbow Surg.

[8] Chisholm C, Poon PC (2012). An in vivo kinematic study of the reverse shoulder joint replacement. Eur J Orthop Surg Traumatol.

[9] Lee KW, Kin YI, Kim HY, Yang DS, Lee GS, Choy WS (2016). Three-dimensional scapular kinematics in patients with reverse total shoulder arthroplasty during arm motion. Clin Orthop Surg.

[10] Matsuki K, Sugaya H, Hoshika S, Takahashi N, Kenmoku T, Banks SA (2018). Scaption kinematics of reverse shoulder arthroplasty do not change after the sixth postoperative month. Clin Biomech.

[11] Roren A, Nguyen C, Palazzo C, Fayad F, Revel M, Gregory T (2017). Kinematic analysis of the shoulder complex after anatomic and reverse total shoulder arthroplasty: a cross-sectional study. Musculoskelet Sci Pract.

[12] Walker D, Matsuki K, Struk AM, Wright TW, Banks SA (2015). Scapulohumeral rhythm in shoulders with reverse shoulder arthroplasty. J Shoulder Elb Surg.

[13] Forte FC, Peduzzi De Castro, de Toledo JM, Ribeiro DC, Loss JF. Scapular kinematics and scapulohumeral rhythm during resisted shoulder abduction—implications for clinical practice. Phys Ther Sport. 2009;10:105–111. 10.1016/j.ptsp.2009.05.005. 10.1016/j.ptsp.2009.05.005 19616180

[14] Kon Y, Nishinaka N, Gamada K, Tsutsui H, Banks SA (2008). The influence of handheld weight on the scapulohumeral rhythm. J Shoulder Elbow Surg.

[15] McQuade KJ, Smidt GL (1998). Dynamic scapulohumeral rhythm: the effects of external resistance during elevation of the arm in the scapular plane. J Orthop Sports Phys Ther.

[16] Michiels I, Grevenstein J (1995). Kinematics of shoulder abduction in the scapular plane. On the influence of abduction velocity and external load. Clin Biomech..

[17] Nishinaka N, Tsutsui H, Mihara K, Suzuki K, Makiuchi D, Kon Y (2008). Determination of in vivo glenohumeral translation using fluoroscopy and shape-matching techniques. J Shoulder Elbow Surg.

[18] Constant CR, Gerber C (2008). A review of the Constant score: modifications and guidelines for its use. J Shoulder Elbow Surg.

[19] Yushkevich PA, Piven J, Hazlett HC, Smith RG, Ho S, Gee JC (2006). User-guided 3D active contour segmentation of anatomical structures: significantly improved efficiency and reliability. Neuroimage.

[20] Banks SA, Hodge WA (1996). Accurate measurement of three-dimentional knee repalacement kinematics using single-plane fluoroscopy. IEEE Trans Biomed Eng.

[21] Koo TK, Li MY (2016). A guideline of selecting and reporting intraclass correlation coefficients for reliability research. J Chiropr Med.

[22] Nobuhara K (2003). The shoulder: its function and clinical aspects.

[23] Mckenna L, Cornwall X, Williams S (2017). Differences in scapular orientation between standing and sitting posture at rest and in 120° scaption: a cross-sectional study. PM R.

[24] Riek LM, Ludewig PM, Nawoczenski DA (2008). Comparative shoulder kinematics during free standing, standing depression lifts and daily functional activities in persons with paraplegia: considerrations for shoulder health. Spinal Cord.

[25] Gulotta LV, Choi D, Marinello P, Knutson Z, Lipman J, Wright T (2012). Humeral component retroversion in reverse total shoulder arthroplasty: a biomechanical study. J Shoulder Elbow Surg.

[26] Matsuki K, Matsuki KO, Yamaguchi S, Ochiai N, Sasho T, Sugaya H (2012). Dynamic in vivo glenohumeral kinematics during scapular plane abduction in healthy shoulders. J Orthop Sports Phys Ther.

[27] Bey MJ, Kline SK, Zauel R, Lock TR, Kolowich PA (2007). Measuring dynamic in vivo glenohumeral joint kinematics: technique and preliminary results. J Biomech.

